# Frailty syndrome in daily practice of interventional cardiology ward—rationale and design of the FRAPICA trial: A STROBE-compliant prospective observational study

**DOI:** 10.1097/MD.0000000000018935

**Published:** 2020-01-31

**Authors:** Ewa Wołoszyn-Horák, Robert Salamon, Katarzyna Chojnacka, Aleksandra Brzosko, Łukasz Bieda, Jakub Standera, Karolina Płoszaj, Ewa Stępień, Ewa Nowalany-Kozielska, Andrzej Tomasik

**Affiliations:** a II Department of Cardiology in Zabrze, Medical Faculty with Dentistry Division in Zabrze, Medical University of Silesia, Katowice; b Dr Teresa Fryda Medical Laboratory, Department in Zabrze; c Department of Medical Physics, Faculty of Physics, Institute of Physics, Astronomy and Applied Computer Science of the Jagiellonian University, Cracow, Poland.

**Keywords:** arterial stiffness, biomarkers, coronary artery disease treatment, fried frailty phenotype score, instrumental activities of daily living score, risk stratification

## Abstract

The effect of frailty on short and long term results of interventional treatment of coronary heart disease is not well defined. The evaluation of frailty may be helpful in appointment of most suitable treatment option and timing of patient follow-up. The frailty syndrome in daily practice of interventional cardiology ward (FRAPICA) study objective is to evaluate prognostic capability of the Fried frailty scale and instrumental activities of daily living scale (IADL) in elderly patients with symptomatic coronary heart disease.

This is a single center, prospective, observational study. Patients aged ≥65 years are eligible. The objectives are to report Fried frailty scale and IADL scale dispersion before hospital discharge and to assess predictive impact of both scores. The endpoints are: success of interventional treatment, its complications (procedure related myocardial infarction, dye-induced renal function deterioration, loss of blood), 3-year mortality, either all-cause and cardiovascular, re-infarction, re-intervention, stroke, new-onset heart failure, any hospital readmission, and a combination of all above mentioned. Secondary analyses will focus on distinct clinical patient presentations, sub-classifications of frailty for modeling of long-term risk.

FRAPICA trial will improve understanding of the associations between frailty syndrome, cardiovascular system diseases, their invasive treatment, and short and long-term outcomes. It will allow for more individualized assessment of risk and will identify new goals for interventions. (ClinicalTrials.gov Identifier NCT03209414)

## 1 Introduction

Frailty gains growing attention of clinical research community as society of developed countries and their cardiovascular patients are aging. Frailty syndrome is characterized by reduced physiological reserve and increased susceptibility to various stressors.^[1]^ Several diseases, either acute or chronic, or iatrogenic circumstances may act as stressors. Frail patients, after being exposed to such stressors, may react with incommensurate decompensation, and adverse outcomes. They are at higher risk of procedural complications, long-lasting recovery, functional deterioration, physical disablement, and increased risk of death.^[2]^ The syndrome is frequently associated with comorbidities.^[3]^ Symptomatic coronary heart disease, either stable or unstable, is the prevailing disease unit among elderly patients. Inversely, data on prevalence of phenotype frailty among patients aged ≥65, who underwent percutaneous coronary angioplasty were recently published.^[4]^ Gharacholou et al^[4]^ reported that approximately 20% of those patients is frail. Moreover, frail patients have greater comorbid burden and greater angiographic coronary artery disease intensity. However, commonly used risk stratification scales, like Syntax,^[5]^ Euro,^[6,7]^ or GRACE^[8]^ use patient's age as a major determinant of risk, but they do not incorporate the frailty traits into the risk assessment. Technical development has allowed more patients to become eligible for interventional treatment.^[9,10]^ As medical innovations are cost consuming, the individual benefits of such medical procedures are not well defined in particular patient populations. Thus, we need to operationalize more detailed patient selection.

The objectives of the study are to report Fried frailty scale and instrumental activities of daily living (IADL) scale dispersion before hospital discharge and to assess the predictive impact of both scores in a population of elderly patients with symptomatic coronary artery disease.

## 2 Materials and methods

### 2.1 Patient population

**Figure 1:**
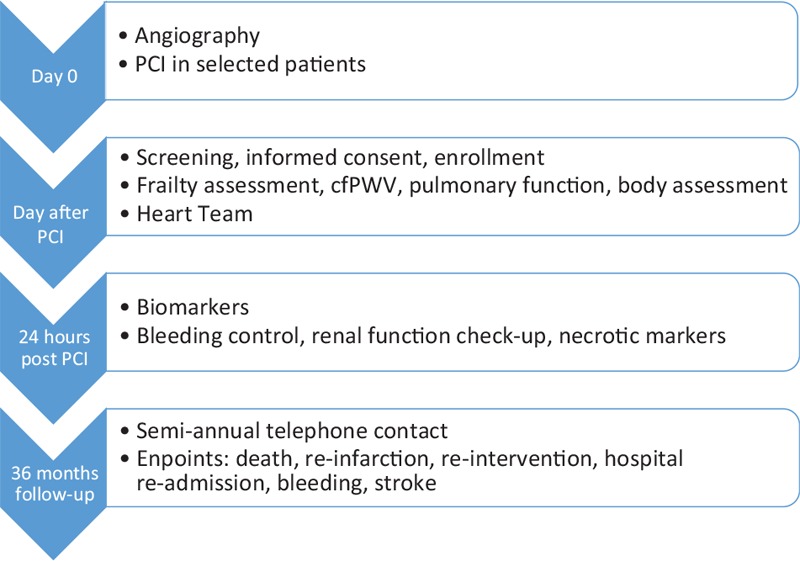
FRAPICA flow chart. FRAPICA = frailty syndrome in daily practice of interventional cardiology ward.

The FRAPICA trial is a single center, observational, prospective, study. One thousand patients aged ≥65 years with symptomatic coronary heart disease will be enrolled. Patients with all clinical presentations of coronary artery disease are eligible. Stable coronary heart disease is diagnosed on the base of exercise induced ischemia, resolving after rest. Acute coronary syndrome includes ST segment elevation myocardial infarction, non-ST segment elevation myocardial infarction and clinical presentation of unstable angina. The latter is characterized by myocardial ischemic pain at rest or at minimal physical activity in the absence of myocardial necrosis. Non-ST segment elevation myocardial infarction is diagnosed in patients with symptoms of ischemia, electrocardiographic abnormalities such as ST-segment depression, inversion of T wave, flattened T wave, pseudo-normalization of previously negative T waves, and presence of cardiomyocyte necrosis. ST-segment elevation myocardial infarction is diagnosed when acute precordial pain (>20 minutes), persistent ST-segment elevation, cardiomyocyte necrosis, and typical angiographic findings are present.^[11]^ Either femoral or radial approaches are acceptable—the choice of puncture site depends on patient's or operator's preference and basically on medical conditions. Only patients with type 1 myocardial infarction are considered eligible for the study. Method of revascularization is decided by the operator and ad hoc percutaneous coronary intervention is sometimes performed. Mostly, this is a case in a low-risk patients with single vessel disease, or in cases of ST-elevation myocardial infarction (STEMI) or non ST-elevation myocardial infarction (NSTEMI) patients with identifiable culprit artery. Nevertheless, all coronary angiographies are assessed and discussed by a heart team, selected records are re-consulted with a cardiac surgeon and a further revascularization schedule is planned. For the risk stratification we use Syntax score and logistic Euro score. After performed coronary angiography and confirmation of coronary heart disease with documented significant stenosis, patients are screened and written informed consent is obtained. Protocol of the study was approved by Internal Review Board of Medical University of Silesia. For the purpose of this study we record demographic, clinical, laboratory, angiographic data from patient's medical charts, assess the presence of frailty syndrome, measure arterial stiffness, body composition, pulmonary function, and draw fasting blood for further analyses. Study flowchart is shown in Fig. 1.

### 2.2 Frailty assessment with Fried frailty scale and IADL score

We use Fried frailty phenotype score^[12]^ and Lawton and Brody score (IADL).^[13]^ We recognize frailty if ≥3 out of 5 following criteria are met^[12]^:

- Slowness—reduced gait speed at a distance of 5 m at usual pace. Patient must repeat 3 times and the results are averaged. If a patient walks for >6 seconds then the criterion is classified positive.
- Weakness is assessed with maximal handgrip strength test. It is carried out in the dominant arm. We use electronic hand dynamometer EH101 (VETEK AB, Sweden). Patient must repeat 3 times and the maximal value is recorded. Test is positive for frailty when strength is lower than 20 kg for women and 30 kg for men.
- Low physical activity is assessed by Minnesota Leisure Time Activity questionnaire. The result is positive when calorie expenditure per week is lower than 270 kcal/wk in women and <383 kcal/wk in men. We have prepared a Microsoft Excel-based template for rapid questioning and easy calculation of all activities and respective calorie expenditure. We are assessing the physical activity from the recent 12 years.
- Exhaustion—self reported by a patient. It is evaluated by the answer to 2 questions from the Center for Epidemiologic Studies Depression Scale Revised (CESD-R) scale. Patient has to answer following questions: “How often in the past week did you feel like everything you did was an effort? How often in the past week did you feel like you could not get going?” The possible answers are: often (≥3 days) or not often, when the feeling is present in 0 to 2 days. Positive answer is when the patient says “often.”
- The last criterion is weight loss weight exceeding 10 pounds (appr. 4.5 kg) unintentionally in the past year.

Patients in whom 1 or 2 criteria are present will be assigned as pre-frail.

The Lawton IADL score is incorporated into CSHA Clinical Frailty Scale for description of mild to severe degrees of frailty.^[14]^ Moreover, even a single deficit in IADL score increases mortality rate by 56% in a cohort of patients with heart failure.^[15]^ We will compare patients with no deficits (IADL score equal to 24 points) with patients with different degrees of impairment (IADL score no >23 points).

### 2.3 Fat-free body mass

Lean body mass is measured using Harpenden skinfold caliper and Baty body assessment software (Baty International Ltd., UK). Lean body mass is derived from patient's height and weight using 3 site Jackson/Pollock algorithm.^[16]^ The algorithm uses different measuring sites for men and women (the sites for male subjects are: chest, abdominal and thigh, for female subjects are: triceps, suprailiac, and thigh). Fat-free body mass is presented as an absolute value and expressed in kilograms, as well as in relation to body weight and expressed in percentage. The inter- and intra-observer variability is 14.4% and 9.1%, respectively.

### 2.4 Pulmonary function assessment

We use Asma-1 (Vitalograph, Ireland) peakflowmeter for assessment of peak expiratory flow (PEF, L/min) and 1 second forced expiratory flow (FEV_1_, L). Test is performed in standing position and we record the highest value out of 3 attempts. Data are presented as actual values and percentages of predicted values. The inter- and intra-observer variability are 9.4% and 7.9%, respectively. There is also a temporal variability up to 19% depending on patient's condition (e.g., patient with chronic obstructive pulmonary diseases).

### 2.5 Pulse wave velocity for arterial stiffness measurement

We use pulse wave velocity between carotid and femoral arteries (cfPWV) for assessment of arterial stiffness. For these measurements we use piezoelectric mechanotransducers in carotid and femoral sites (Complior, Alam Medical, France). This methodology is recommended by European Society of Hypertension.^[17]^ The right-sided carotid-femoral distance is measured with Seca mod. 207 height meter (Seca, Germany). Blood pressure is measured in a supine position, after at least 5 minutes of rest using Microlife BP A1 sphygmomanometer immediately before PWV assessment on a dominant arm. Arterial stiffness is expressed in meters per second. We will analyze also derived variables like: central blood pressure, central pulse pressure, and augmentation index. Derivatives are calculated by the software from the carotid pulse waveform. The inter- and intra-observer coefficients of variation are 12.3% and 7.4%, respectively.

### 2.6 Biomarkers

Twenty milliliters of fasting blood is drawn for preparation of blood plasma, serum, and peripheral blood mononuclear cells. Samples are frozen at –85 °C until further analysis. All analyses will be done after enrollment of the last patient. We plan to measure following biomarkers: C-reactive protein, tumor necrosis alpha (TNFα), interleukin 6 (IL-6), soluble suppression of tumorigenicity protein (ST2), klotho, galectin3, pregnancy associated plasma protein A (PAPP-A), telomere length and telomerase activity, specific miRNA (e.g., mir-122-5p, miR-126, miR-133, miR-16). Soluble ST2 will be measured using Aspect-Plus ST2 Test (Critical Diagnostics, California, USA). Its sensitivity limit is 12.5 ng/mL, recommended upper reference limit is 35 ng/mL. Total coefficient of variation is 14.2%.

### 2.7 Study outcomes

The outcomes are designed to answer the following questions: effectiveness of revascularization, its safety, and the overall health of study population in 36-month long follow-up. We consider effective revascularization as complete supply of blood to all ischemic territories of coronary tree, obtained either percutaneously or surgically. In some cases of multi-vessel coronary heart disease and percutaneous revascularization we allow the revascularization to be performed in staged procedures. In such cases, the effectiveness will be assessed after final procedure. The assessment will be carried out by 2 independent operators by visual evaluation of final angiogram. Any discrepancies in assessment will be resolved by consensus. Any functional or alternative imaging assessments, like intravascular ultrasound, optical coherence tomography, or fractional flow reserve are left for operator's decision and will be added to our records.

In the category of procedural safety we will assess peri-interventional myocardial infarction, contrast induced nephropathy, and bleeding. Periprocedural myocardial infarction is defined according to description provided by Thygesen et al.^[18]^ It is characterized as “elevation of cardiac troponins >5× 99th percentile of upper reference limit occurring within 48 hours of the procedure plus either evidence of prolonged ischemia (20 minutes) as demonstrated by prolonged chest pain, or ischemic ST changes or new pathological Q waves, or angiographic evidence of a flow limiting complication, such as of loss of patency of a side branch, persistent slow-flow or no-reflow, embolization, or imaging evidence of new loss of viable myocardium or new regional wall motion abnormality.”

Contrast induced nephropathy is defined after Parfrey et al^[19]^ as “increase in the serum creatinine concentration of >25%, or of >0.5 mg/dL within 48 hours after the administration of the contrast agent.”

Bleeding is defined after Smith et al^[20]^ as “blood loss at the site of arterial or venous access or due to perforation of a traversed artery or vein requiring transfusion and/or prolonging the hospital stay, and/or causing a drop in hemoglobin >3.0 g/dL. Bleeding attributable to the vascular site could be retroperitoneal, a local hematoma >10 cm diameter or external.” Bleeding from non-access sites like intracranial hemorrhage, gastrointestinal, or genitourinary blood loss will be recorded either to ascertain safety of antiplatelet or anticoagulation treatment in frail patients.

For the assessment of overall patients’ health we will monitor 3-year mortality, either all-cause and cardiovascular, re-infarction, re-intervention, stroke, new-onset heart failure, any hospital readmission, and a combination of all above mentioned. The 36-months long follow-up is based on semi-annual telephone contact with patient or designated family member.

### 2.8 Risk stratification and treatment planning tools

The leading rules used in designing the treatment plan are these described in European Society of Cardiology respective guidelines.^[11,21,22]^ For precise risk stratification we use the following scores:

- Syntax score I and II—www.syntaxscore.com^[5]^;
- Logistic Euroscore 2—www.euroscore.org^[6]^;
- GRACE 2.0 score—www.gracescore.org^[23]^;
- CRUSADE bleeding risk score—www.crusadebleedingscore.org.^[24]^

The final results of each score will be compared between frail, pre-frail, and non-frail patients and will be used for modeling of the outcomes hazard ratio in the follow-up.

### 2.9 Echocardiographic assessment of left ventricle geometry and function

We assess left ventricle diameters, volumes, systolic and diastolic function according to American Society of Echocardiography guidelines for chamber quantification.^[25]^ We use Vivid 7 Dimension echocardiographic machine with integrated software (GE, Horten, Norway) for measurements.

### 2.10 Statistical analysis

First, we use descriptive and comparative statistical methods to describe the incidence of frailty among symptomatic patients with coronary artery disease and provide extensive characteristics of frail elderly coronary heart disease population. Two-way analyses with *P* < .05 are considered significant. Second, Kaplan–Meier analysis of survival and Cox's proportional hazard analysis will be used for the identification of variables predisposing for outcomes occurrence. Selection of appropriate variables will be based on results of comparative and descriptive statistical analysis. Variables of significance <0.1 will be entered into uni- and multivariate Cox modeling.

**Figure 2:**
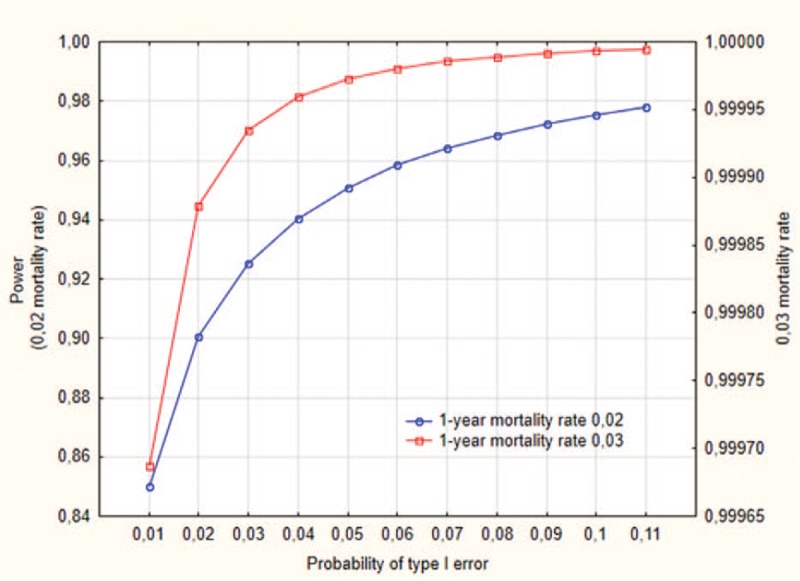
A plot of study power versus type I error for the sample size of 1000 patients and 2 different 1-year mortality rates.

According to the data reported by Central Statistical Office of Poland (www.stat.gov.pl), in 2013 the number of deaths from cardiovascular diseases ranged from 2226 to 3086 per 100,000 inhabitants over 65 years of age. These data allow to predict the occurrence of approximately 100 deaths in the study population during 36 months of prospective observation. Power of study based on the mortality data is presented in Fig. 2. The other endpoint is the incidence of new-onset heart failure. In our observations (unpublished data), the incidence of symptomatic heart failure in STEMI-treated patients after myocardial infarction was 16.3% in 12 months. Considering that patients with STEMI infarction will be around 100, we assume at least 45 new episodes of cardiac failure over a 36-month period. These assumptions were necessary for reliable Cox proportional hazard modeling—10 complete observations are required for a reliable estimation of 1 variable. The ancillary analyses will be performed for different subgroups depending on demographic or clinical presentations and we will analyze either crude or adjusted data.^[26]^ All analyses will be performed in Statistica 12.5 (Tulsa, Oklahoma, OK) licensed to Medical University of Silesia.

## 3 Expected results and possible pitfalls

We expect to obtain results which will be evaluable in 3 categories: cross-sectional, baseline demographics, short-term longitudinal results of interventional treatment, and long-term follow-up results on adverse cardiovascular outcomes. We hope that we will be able to distinguish several clinical groups of patients, according to frailty status and relevant to procedural risk, like low-risk, medium-risk, and high-risk, or relevant to patient's clinical benefit. The major possible pitfalls of the study, that we must be aware of, are as follows. Our study is a single center, thus the extension of our results to community dwelling population or patients of other races will require discussion. Second, we are recruiting coronary artery disease patients only, but substantial proportion of them will have concomitant heart failure. Heart failure associated diminished functional capacity may mimic or be a true component of frailty. Heart failure in frail elderly patients may be considered as inseparable triangle.^[27]^ At the same time, active angina may also limit patients’ gait speed and result in over-diagnosing of frailty. In the population of truly pre-frail patients, stressors such as disease exacerbations, or hospital admission may result in dynamic transition to frail status. Thus, we must be aware of primary and secondary frailty, and its dynamic nature.^[27,28]^ We have diligently applied Fried frailty score as the most evaluator-independent and reproducible tool to assess frailty and compare its distribution with other populations.

## 4 Discussion

We do hope that FRAPICA trial with its prospective, observational design, and control of many variables will clarify several issues and the results will be readily transferable for clinical practice. We use 2 different tools for frailty assessment: Fried frailty scale requires accessory equipment (hand dynamometer, stopwatch, computer for leisure time activity calculation) for patient evaluation, while IADL score is ready for use equipment-free assessing tool. Each of the tools will be used for risk stratification separately and in combination. Their predictive value will be compared at the same time. Apart from these scales, we have several additional variables which will be used for supplementary characteristics of frail elderly patients and will be integrated into risk stratification model enabling decision making in this population of patients. The clinical data which we assume may prove important for risk assessment would be evaluated routinely as an evidence-based daily clinical practice. To our knowledge there are only few previous publications dealing with results of interventional treatment.^[4,29]^ The researchers from Mayo Clinic have used the same Fried phenotype frailty assessment tool, but have limited the study population to elderly patients who underwent percutaneous coronary intervention. We aim to analyze 2 other patients’ populations either: this treated surgically and this treated medically.

Complimentary measures of cardiovascular status and body composition will be helpful in better characterization of frailty. Arterial stiffness is more and more often distinguished as a measure of cardiovascular status.^[30]^ It may precipitate symptoms of angina with insignificant lesions within coronary arteries, or it may lead to occurrence of diastolic heart failure in elderly patients.^[31]^ Measurement of arterial stiffness, as a simple and a non-invasive method, can be easily applied at patient's bedside.^[30]^ Of note, increased pulse wave velocity in elderly patients indicates increased risk of adverse cardiovascular outcomes.^[32]^

Mechanisms implicated in the development of frailty syndrome include impaired function of the immunological,^[33]^ hormonal,^[34]^ and endocrine networks.^[35]^ This results in a formation of catabolic environment, in which muscle decomposition dominates, ending up eventually in sarcopenia.^[36]^ We suppose that extensive data on patients’ lean body mass, left ventricle mass, geometry, and function, will allow to elucidate further the phenotype of frailty. It is of note, that we will have the data on elderly patients with symptomatic coronary heart disease—a sample completely different from a community-based population.

## 5 Conclusion

FRAPICA trial will improve understanding of the associations between frailty syndrome, cardiovascular system diseases, their invasive treatment and short and long-term outcomes. It will allow for more individualized assessment of risk and will identify new goals for interventions.

## Author contributions

**Conceptualization:** Ewa Wołoszyn-Horák, Robert Salamon, Katarzyna Chojnacka, Aleksandra Brzosko, Łukasz Bieda, Jakub Standera, Ewa Stępień, Ewa Nowalany-Kozielska, Andrzej Tomasik.

**Data curation:** Ewa Wołoszyn-Horák, Robert Salamon, Katarzyna Chojnacka, Aleksandra Brzosko, Łukasz Bieda, Jakub Standera, Ewa Stępień, Ewa Nowalany-Kozielska, Andrzej Tomasik.

**Formal analysis:** Ewa Wołoszyn-Horák, Robert Salamon, Katarzyna Chojnacka, Aleksandra Brzosko, Łukasz Bieda, Jakub Standera, Karolina Płoszaj, Ewa Stępień, Ewa Nowalany-Kozielska, Andrzej Tomasik.

**Funding acquisition:** Andrzej Tomasik.

**Investigation:** Ewa Wołoszyn-Horák, Robert Salamon, Katarzyna Chojnacka, Aleksandra Brzosko, Łukasz Bieda, Jakub Standera, Karolina Płoszaj, Ewa Stępień, Andrzej Tomasik.

**Methodology:** Ewa Wołoszyn-Horák, Robert Salamon, Aleksandra Brzosko, Łukasz Bieda, Jakub Standera, Karolina Płoszaj, Ewa Nowalany-Kozielska, Andrzej Tomasik.

**Project administration:** Andrzej Tomasik.

**Resources:** Karolina Płoszaj, Andrzej Tomasik.

**Software:** Andrzej Tomasik.

**Supervision:** Ewa Stępień, Ewa Nowalany-Kozielska, Andrzej Tomasik.

**Validation:** Karolina Płoszaj, Andrzej Tomasik.

**Visualization:** Andrzej Tomasik.

**Writing – original draft:** Ewa Wołoszyn-Horák, Robert Salamon, Katarzyna Chojnacka, Aleksandra Brzosko, Łukasz Bieda, Jakub Standera, Karolina Płoszaj, Ewa Stępień, Ewa Nowalany-Kozielska, Andrzej Tomasik.

**Writing – review & editing:** Ewa Stępień, Ewa Nowalany-Kozielska, Andrzej Tomasik.

Andrzej Tomasik orcid: 0000-0002-9312-762X.

Ewa Stępień orcid: 0000-0003-3589-1715.

Ewa Nowalany-Kozielska orcid: 0000-0002-5977-513X.

## References

[1] Bergman H Ferrucci L Guralnik J Frailty: an emerging research and clinical paradigm--issues and controversies. J Gerontol A Biol Sci Med Sci 2007;62:731–7. 17634320 10.1093/gerona/62.7.731 PMC2645660

[2] Shamliyan T Talley KM Ramakrishnan R Association of frailty with survival: a systematic literature review. Ageing Res Rev 2013;12:719–36. 22426304 10.1016/j.arr.2012.03.001

[3] Fulop T Larbi A Witkowski JM Aging, frailty and age-related diseases. Biogerontology 2010;11:547–63. 20559726 10.1007/s10522-010-9287-2

[4] Gharacholou SM Roger VL Lennon RJ Comparison of frail patients versus nonfrail patients >/=65 years of age undergoing percutaneous coronary intervention. Am J Cardiol 2012;109:1569–75. 22440119 10.1016/j.amjcard.2012.01.384 PMC5018988

[5] Kappetein AP Dawkins KD Mohr FW Current percutaneous coronary intervention and coronary artery bypass grafting practices for three-vessel and left main coronary artery disease. Insights from the SYNTAX run-in phase. Eur J Cardiothorac Surg 2006;29:486–91. 16497510 10.1016/j.ejcts.2006.01.047

[6] Roques F Michel P Goldstone AR The logistic EuroSCORE. Eur Heart J 2003;24:881–2. 10.1016/s0195-668x(02)00799-6 12727160

[7] Roques F Nashef SA Michel P Risk factors and outcome in European cardiac surgery: analysis of the EuroSCORE multinational database of 19030 patients. Eur J Cardiothorac Surg 1999;15:816–22. discussion 822-3. 10431864 10.1016/s1010-7940(99)00106-2

[8] Yan AT Yan RT Tan M In-hospital revascularization and one-year outcome of acute coronary syndrome patients stratified by the GRACE risk score. Am J Cardiol 2005;96:913–6. 16188515 10.1016/j.amjcard.2005.05.046

[9] Page M Doucet M Eisenberg MJ Temporal trends in revascularization and outcomes after acute myocardial infarction among the very elderly. CMAJ 2010;182:1415–20. 20682731 10.1503/cmaj.092053 PMC2942913

[10] Dodson JA Maurer MS Changing nature of cardiac interventions in older adults. Aging health 2011;7:283–95. 21743812 10.2217/ahe.11.12 PMC3129702

[11] Roffi M Patrono C Collet J-P 2015 ESC Guidelines for the management of acute coronary syndromes in patients presenting without persistent ST-segment elevationTask Force for the Management of Acute Coronary Syndromes in Patients Presenting without Persistent ST-Segment Elevation of the European Society of Cardiology (ESC). Eur Heart J 2016;37:267–315. 26320110 10.1093/eurheartj/ehv320

[12] Fried LP Tangen CM Walston J Frailty in older adults: evidence for a phenotype. J Gerontol A Biol Sci Med Sci 2001;56:M146–56. 11253156 10.1093/gerona/56.3.m146

[13] Lawton MP Brody EM Assessment of older people: self-maintaining and instrumental activities of daily living. Gerontologist 1969;9:179–86. 5349366

[14] Rockwood K Song X MacKnight C A global clinical measure of fitness and frailty in elderly people. CMAJ 2005;173:489–95. 16129869 10.1503/cmaj.050051 PMC1188185

[15] Lo AX Donnelly JP McGwin G Jr Impact of gait speed and instrumental activities of daily living on all-cause mortality in adults >/=65 years with heart failure. Am J Cardiol 2015;115:797–801. 25655868 10.1016/j.amjcard.2014.12.044 PMC4474480

[16] Jackson AS Pollock ML Practical assessment of body composition. Phys Sportsmed 1985;13:76–90. 10.1080/00913847.1985.11708790 27463295

[17] Mancia G Fagard R Narkiewicz K 2013 ESH/ESC guidelines for the management of arterial hypertension: the Task Force for the Management of Arterial Hypertension of the European Society of Hypertension (ESH) and of the European Society of Cardiology (ESC). Eur Heart J 2013;34:2159–219. 23771844 10.1093/eurheartj/eht151

[18] Thygesen K Alpert JS Jaffe AS Third universal definition of myocardial infarction. Eur Heart J 2012;33:2551–67. 22922414 10.1093/eurheartj/ehs184

[19] Parfrey PS Griffiths SM Barrett BJ Contrast material-induced renal failure in patients with diabetes mellitus, renal insufficiency, or both. N Engl J Med 1989;320:143–9. 2643041 10.1056/NEJM198901193200303

[20] Smith SC Jr Dove JT Jacobs AK ACC/AHA guidelines of percutaneous coronary interventions (revision of the 1993 PTCA guidelines)--executive summary. A report of the American College of Cardiology/American Heart Association Task Force on Practice Guidelines (committee to revise the 1993 guidelines for percutaneous transluminal coronary angioplasty). J Am Coll Cardiol 2001;37:2215–39. 11419905 10.1016/s0735-1097(01)01344-4

[21] Ibanez B James S Agewall S 2017 ESC Guidelines for the management of acute myocardial infarction in patients presenting with ST-segment elevation. The Task Force for the management of acute myocardial infarction in patients presenting with ST-segment elevation of the European Society of Cardiology (ESC). Eur Heart J 2018;39:119–77. 28886621 10.1093/eurheartj/ehx393

[22] Windecker S Kolh P Alfonso F Authors/Task Force m. 2014 ESC/EACTS Guidelines on myocardial revascularization: The Task Force on Myocardial Revascularization of the European Society of Cardiology (ESC) and the European Association for Cardio-Thoracic Surgery (EACTS) developed with the special contribution of the European Association of Percutaneous Cardiovascular Interventions (EAPCI). Eur Heart J 2014;35:2541–619. 25173339 10.1093/eurheartj/ehu278

[23] Devlin G Gore JM Elliott J Management and 6-month outcomes in elderly and very elderly patients with high-risk non-ST-elevation acute coronary syndromes: The Global Registry of Acute Coronary Events. Eur Heart J 2008;29:1275–82. 18387940 10.1093/eurheartj/ehn124

[24] Yang X Alexander KP Chen AY The implications of blood transfusions for patients with non-ST-segment elevation acute coronary syndromes: results from the CRUSADE National Quality Improvement Initiative. J Am Coll Cardiol 2005;46:1490–5. 16226173 10.1016/j.jacc.2005.06.072

[25] Lang RM Bierig M Devereux RB Recommendations for chamber quantification: a report from the American Society of Echocardiography's Guidelines and Standards Committee and the Chamber Quantification Writing Group, developed in conjunction with the European Association of Echocardiography, a branch of the European Society of Cardiology. J Am Soc Echocardiogr 2005;18:1440–63. 16376782 10.1016/j.echo.2005.10.005

[26] Snedecor G Cochrane W Statistical Methods. VII ed. Iowa; 1980.

[27] Shinmura K Cardiac senescence, heart failure, and frailty: a triangle in elderly people. Keio J Med 2016;65:25–32. 27170235 10.2302/kjm.2015-0015-IR

[28] Morley JE Vellas B van Kan GA Frailty consensus: a call to action. J Am Med Dir Assoc 2013;14:392–7. 23764209 10.1016/j.jamda.2013.03.022 PMC4084863

[29] Singh M Rihal CS Lennon RJ Influence of frailty and health status on outcomes in patients with coronary disease undergoing percutaneous revascularization. Circ Cardiovasc Qual Outcomes 2011;4:496–502. 21878670 10.1161/CIRCOUTCOMES.111.961375 PMC4182923

[30] Laurent S Cockcroft J Van Bortel L Expert consensus document on arterial stiffness: methodological issues and clinical applications. Eur Heart J 2006;27:2588–605. 17000623 10.1093/eurheartj/ehl254

[31] Weber T Auer J O’Rourke MF Prolonged mechanical systole and increased arterial wave reflections in diastolic dysfunction. Heart 2006;92:1616–22. 16709696 10.1136/hrt.2005.084145 PMC1861240

[32] Veerasamy M Ford GA Neely D Association of aging, arterial stiffness, and cardiovascular disease: a review. Cardiol Rev 2014;22:223–32. 24441048 10.1097/CRD.0000000000000009

[33] Walston J McBurnie MA Newman A Frailty and activation of the inflammation and coagulation systems with and without clinical comorbidities: results from the Cardiovascular Health Study. Arch Intern Med 2002;162:2333–41. 12418947 10.1001/archinte.162.20.2333

[34] Travison TG Nguyen AH Naganathan V Changes in reproductive hormone concentrations predict the prevalence and progression of the frailty syndrome in older men: the concord health and ageing in men project. J Clin Endocrinol Metab 2011;96:2464–74. 21677041 10.1210/jc.2011-0143

[35] Barzilay JI Blaum C Moore T Insulin resistance and inflammation as precursors of frailty: the Cardiovascular Health Study. Arch Intern Med 2007;167:635–41. 17420420 10.1001/archinte.167.7.635

[36] BoirieY Physiopathological mechanism of sarcopenia. J Nutr Health Aging 2009;13:717–23.1965755610.1007/s12603-009-0203-x

